# Modulation of long-chain Acyl-CoA synthetase on the development, lipid deposit and cryosurvival of *in vitro* produced bovine embryos

**DOI:** 10.1371/journal.pone.0220731

**Published:** 2019-08-05

**Authors:** Roniele Santana Valente, Tamie Guibu de Almeida, Mayra Fernanda Alves, Janine de Camargo, Andrea Cristina Basso, Katia Roberta Anacleto Belaz, Marcos Nogueira Eberlin, Fernanda da Cruz Landim-Alvarenga, Patricia Kubo Fontes, Marcelo Fábio Gouveia Nogueira, Mateus José Sudano

**Affiliations:** 1 School of Veterinary Medicine, Federal University of Pampa, Uruguaiana, RS, Brazil; 2 Center for Natural and Human Sciences, Federal University of ABC, Santo André, SP, Brazil; 3 Department of Animal Reproduction, University of São Paulo, São Paulo, SP, Brazil; 4 In Vitro Brasil, ABS Pecplan, Mogi Mirim, SP, Brazil; 5 Department of Chemistry, Federal University of Uberlândia, Uberlândia, MG, Brazil; 6 School of Engineering, Mackenzie Presbyterian University, São Paulo, SP, Brazil; 7 Department of Animal Reproduction, São Paulo State University, Botucatu, SP, Brazil; 8 Department of Biological Sciences, São Paulo State University, Assis, SP, Brazil; University of Florida, UNITED STATES

## Abstract

In this study, we evaluated the modulation effect of long-chain Acyl-CoA synthetase during early embryo development. Bovine embryos were cultured in four groups: positive modulation (ACS+) with GW3965 hydrochloride, negative modulation (ACS-) with Triacsin C, association of both modulators (ACS±), and control. Embryo development rates were not altered (P>0.05) by treatments. Embryonic cytoplasmic lipid content increased in ACS+ but reduced in ACS- compared to the control (P < 0.05), whereas the membrane phospholipids profile was not altered by treatments. The total number of blastomeres did not differ (P > 0.05) between groups; however, an increased apoptotic cells percentage was found in ACS- compared to control. Twenty-four hours after warming, ACS+ and control grade I embryos presented the best hatching rates, whereas the ACS+ group equaled the hatching rates between their embryos of grades I, II and III 48 hours after warming. The relative abundance of transcripts for genes associated with lipid metabolism (*ACSL3*, *ACSL6*, *ACAT1*, *SCD*, and *AUH*), heatshock (*HSP90AA1* and *HSF1*), oxidative stress (*GPX4*), and angiogenesis (*VEGF*), among other important genes for embryo development were affected by at least one of the treatments. The treatments were effective in modulating the level of transcripts for *ACSL3* and the cytoplasmic lipid content. The ACS- was not effective in increasing embryonic cryosurvival, whereas ACS+ restored survival rates after vitrification of embryos with low quality, making them equivalent to embryos of excellent quality.

## Introduction

Over the past several decades, increased demand for *in vitro* produced (IVP) bovine embryos has triggered a constant search for improvements of the technique, aiming at the production of embryos of better quality that result in higher pregnancy rates. Despite being considered an already established biotechnology, the high sensitivity after cryopreservation still represents one of the greatest challenges to be overcome for the wide dissemination of IVP embryo transfer. In view of the satisfactory results obtained with embryos produced *in vivo*, the reduced cryosurvival of IVP may be directly related to sub-optimal *in vitro* culture conditions during development, leading to structural and functional modifications that compromise embryo viability.

In recent years, the existence of morphological and metabolic differences in IVP embryos has been clearly demonstrated in relation to *in vivo*-produced ones. Among these differences is the higher cytoplasmic lipid content, commonly associated with lower rates of embryonic survival after freezing [1] and deviations in the relative abundance of transcripts of important genes for embryonic development and establishment of gestation [2].

The mechanisms through which IVP embryos accumulate more lipids are not yet fully elucidated; however, these embryos are known to be less resistant to cryopreservation when compared to those produced *in vivo*. Alternatives such as changes in media culture conditions, like the addition of lipolytic chemicals [3] or the reduction or removal of fetal calf serum [4–6] were proposed to decrease the amount of lipid droplets within the embryonic cytoplasm.

The participation of fatty acids in most metabolic pathways, including β-oxidation and biosynthesis of complex lipids (such as triacylglycerols and phospholipids), requires their initial activation by the addition of a CoA group [7] resulting in the formation of Acyl-CoA, a reaction catalyzed by Acyl-CoA synthetase (ACS) [8]. Three ACS subfamilies are expressed in mammals: long chain acyl-CoA synthetase (ACSL), fatty acid transport proteins (FATP) and Acyl-CoA synthetase bubblegum (ACSBG) [9]. Five genes of the ACSL family were identified based on sequence homology, tissue and intracellular distribution, and termed *ACSL1* and *ACSL3* to *ACSL6*.

Liver nuclear X receptors α and β (LXRα and LXRβ) are regulators of lipid metabolism in many tissues, acting on the expression of multiple genes involved in the cholesterol efflux, transport and excretion, fatty acid biosynthesis, and lipoprotein metabolism in different tissues [10]. Triacsin C is a fungal metabolic regulator that acts as a competitive selective inhibitor of ACLS 1, 3 and 4 [11] almost completely inhibiting the *de novo* synthesis of triacylglycerol and phospholipids from glycerol in human fibroblasts [12]. GW3965 hydrochloride is an agonist specific for liver X receptors (LXRα and LXRβ), and is capable of inducing an increase in the expression of hepatic *ACSL3* in hamsters [13] and placental trophoblast cells [7]. Apparently, to date there are no reports of the action of these compounds on embryonic development.

The objective of the present work was to evaluate the effects of the addition of GW3965 hydrochloride and Triacsin C, positive (ACS+) and negative (ACS-) modulators of ACSL, respectively, on development, cryosurvival, lipid accumulation and profile, gene expression, and viability of bovine IVP embryos.

## Material and methods

### Reagents

All materials were acquired from Sigma (Sigma-Aldrich Corp.) except when specified.

### Experimental design

The positive and negative modulators of ACSL, GW3965 hydrochloride (ACS+) and Triacsin C (ACS-), were added to the *in vitro* culture medium of bovine embryos. On the fourth day of culture (96 hpi) the embryos were randomly distributed into four groups: ACS+, ACS-, association between modulators (ACS±) or control group. The procedure consisted of the withdrawal of 2.5 μl of culture medium and addition of 2.5 μl of the modulator in each group. Day four was selected to start the treatment because is the moment that precede: i) the highest lipid content of the total embryo area at morula stage (not normalized by cell number), ii) the drop of lipid content from morula to blastocyst (normalize or not by the embryo cell number), and iii) the shift in membrane lipid profile during early embryo development [14, 15].

Production rates and embryo quality were recorded. Embryo re-expansion and hatching rates were evaluated at 12, 24, and 48 hours after warming. The total number of cells and the apoptotic cells index were measured for evaluation of embryonic viability. In addition, the cytoplasmic lipid droplets content (Sudan Black B) and membrane phospholipid profiles (MALDI-MS) were determined. Finally, the expression of genes related to lipid metabolism and embryonic quality were investigated.

### Pilot study

To test the most appropriate dose of each modulator to be used, a pilot study was performed using 1100 oocytes in which the concentrations of 100, 10, and 1 x (literature reference dose), 10^−1^ x and 10^−2^ x of each drug were tested in relation to the control group. On the fourth day of culture, embryos were randomly distributed among the groups that would receive each of the doses of ACS+, ACS-, or to the control group. The highest dose of each modulator that did not significantly affect the production and quality of the embryos in relation to the control group was selected (GW3965 hydrochloride 10 x (10 μM) and Triacsin C 10^−2^ x (0.1 μM) and used in the next replicas. The protocol for the embryos production is described below.

### *In vitro* recovery and maturation (IVM) of cumulus-oocyte complexes

Bovine ovaries (predominantly *Bos taurus indicus*) were collected at a local slaughterhouse and kept in saline solution heated to 35°C until use. Antral follicles (2 to 8 mm in diameter) were aspirated using an 18G needle and 10 mL syringe. A total of 3,393 cumulus-oocyte complexes (COCs) containing homogeneous cytoplasm and at least three layers of cumulus cells were selected and incubated at 38.5°C in 5% CO_2_ atmosphere and 100% humidity for 24 h. The maturation process was carried out in groups of 20–25 COCs, in drops containing 90 μL of serum-containing (10% v/v) IVM medium (*In Vitro* Brazil—IVB | ABS Pecplan), placed in Petri dishes, and covered with mineral oil.

### *In vitro* fertilization (IVF) of oocytes

At the end of the maturation period, the COCs (20–25) were transferred to new Petri dishes containing 90 μL of fertilization medium (*In Vitro* Brazil—IVB | ABS Pecplan). For *in vitro* fertilization (IVF), a sperm pool from the commercial semen of four different bulls (two Nellore, one Gyr and one Holstein bulls) with proven fertility was used. Viable sperm was selected using a Percoll gradient [16] and IVF was performed with 2 × 10^6^ spermatozoa/mL. Co-culture was maintained at a temperature of 38.5°C in 5% CO_2_ atmosphere and 100% humidity for 18 h. In total, four batches of IVF were performed.

### *In vitro* culture (IVC) of embryos

After the IVF period, the presumptive zygotes were denuded by successive pipetting and then transferred to Petri dishes containing 100 μL of mSOF culture medium (*In Vitro* Brazil—IVB | ABS Pecplan) supplemented with 5 mg/mL of BSA and 2.5% (v/v) of fetal calf serum, covered with mineral oil and maintained at a temperature of 38.5°C in an atmosphere of 5% CO_2_, 5% O_2_, and balance of N_2_ with 100% of humidity. A serum-supplemented medium was used because: *i*) it is the standard *in vitro* culture media used for majority *in vitro* production of bovine embryos worldwide; ii) there is evidences that increased activity of ACSL3 is associated with the fatty acid uptake [7]; and iii) to try to pharmacology modulate lipid content and cryosurvival of the IVP embryos in serum containing medium. On day four (D4–96hpi), prior to the treatment with the modulators and in order to avoid an additional petri dish handling, 50% of the culture medium was replaced with the same amount of new mSOF plus glucose [1 μg/mL]. The embryos were then randomly distributed between the groups that received the treatments with individual modulators (ACS+, 10 μM), (ACS-, 0,1 μM), association of both modulators (ACS±; ACS+ 10 μM and ACS- 0,1 μM)) or the control group. Each modulator used was diluted in dimethylsulfoxide (DMSO) and rediluted in PBS, according manufacturer’s instructions. The procedure consisted of the removal of 2.5 μL of culture medium and addition of 2.5 μL of the respective modulator in each of the groups. The vehicle used to dilute the modulators was added to the control group (2.5 μL of PBS). Cleavage and blastocyst rates were recorded on day two and day 8. For all analyses, embryos were collected at the expanded blastocyst stage on D6, D7 and D8. Expanded blastocyst quality was evaluated based on embryonic mass symmetry, size, color, and uniformity of the blastomeres and classified morphologically as grade I, II and III, according to methodology proposed by Stringfellow and Seidel [17].

### Vitrification of embryos

All embryos in the expanded blastocyst stage (n = 293) were cryopreserved by the Cryotop vitrification method, as described by Sanches, Marinho [18]. Briefly, grade I, II and III embryos were equilibrated in base medium (TCM-HEPES + 20% fetal calf serum) and then transferred to base medium supplemented with 10% ethylene glycol and 10% DMSO for 1 minute. The embryos were then transferred to a vitrification solution consisting of 20% ethylene glycol, 20% DMSO, and 0.5 M sucrose in base medium, and incubated for 20 seconds. They were then deposited on the top of a polypropylene strip of a Cryotop [19] (three to five embryos) with a minimal amount of vitrification solution, and then immediately immersed in liquid nitrogen (N_2_).

### Warming and re-culture of embryos

For warming, cryotops containing the embryos were withdrawn from the N_2_, held for four seconds at room temperature and immediately immersed in TCM-HEPES medium + 20% fetal calf serum + 0.5M sucrose at 35°C where they remained for one minute. Then, the embryos were transferred to TCM-HEPES media + 20% SFB + 0.3M sucrose and TCM-HEPES + 20% FBS + 0.15M sucrose, at room temperature, where they remained for five minutes on each solution. The embryos were washed in mSOF medium and cultured under the same conditions described above during a period of 48 h. Embryo cryosurvival was defined based on the blastocoel re-expansion after 12 hours, followed by total hatching of the zona pellucida evaluated at 24 and 48 hours of incubation.

### Semiquantitative and semimorphometric evaluation of cytoplasmic lipid droplets content

Expanded blastocysts (N = 18 per group) were initially fixed in 10% formaldehyde solution for 2 hours at room temperature. Subsequently, they were transferred to 50% ethanol droplets and then placed in drops of 1% Sudan-Black B (cytoplasmic lipophilic dye) diluted in 70% ethanol for 2 min. The embryos were then washed in 50% ethanol drops and finally placed on slides containing glycerol and covered with coverslips. The analysis was performed under an optical microscope at a magnification of 200 x. The semiquantitative lipid droplets content was estimated using Image J 1.4 software. Images captured of the embryos were converted to a gray scale. The gray intensity mean was recorded and the area of the embryo was calculated with the use of the freehand selection tool. The semiquantitative lipid content data is presented as gray intensity mean per area (gray intensity/μm^2^).

**Fig 1:**
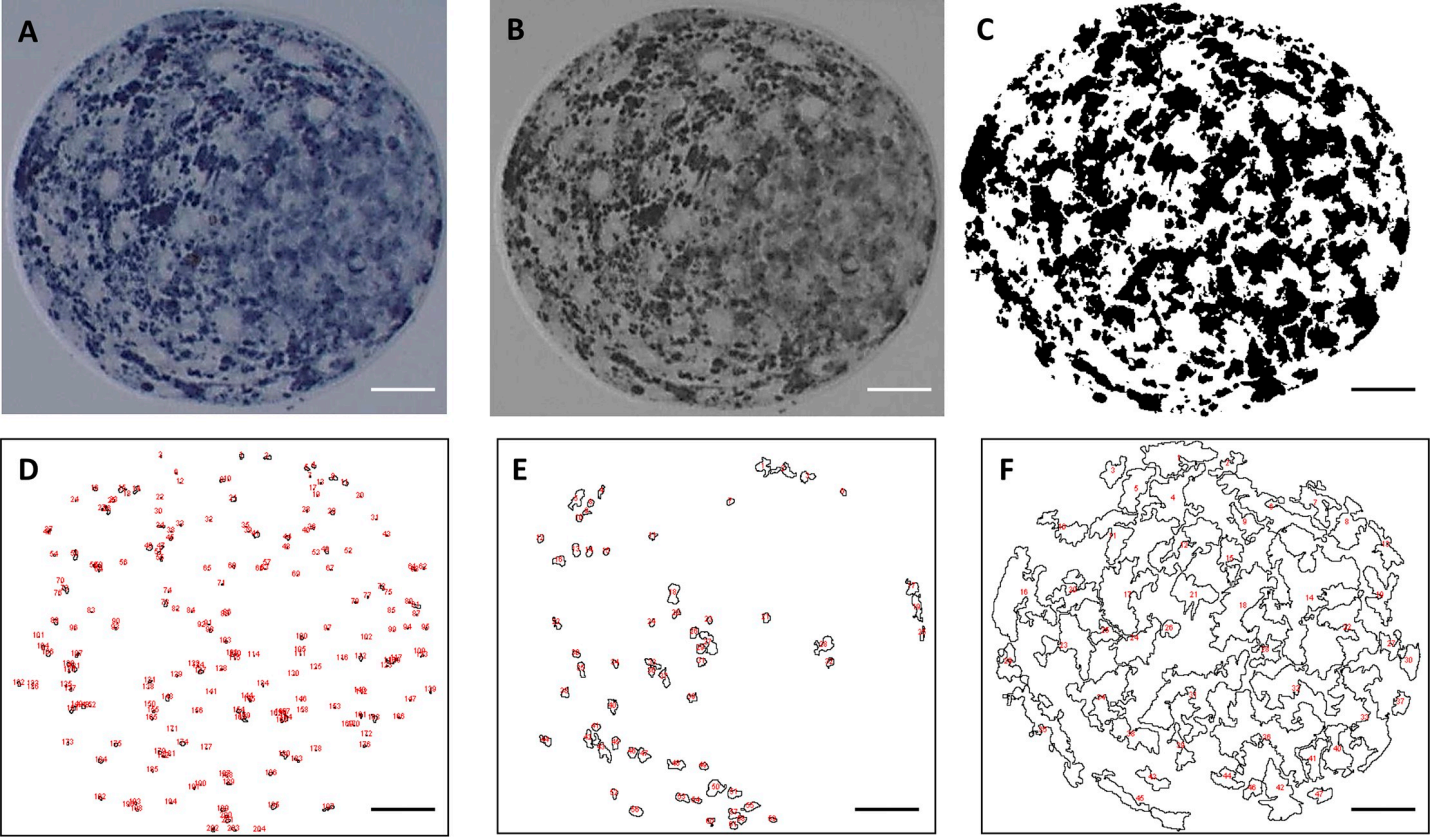
A representative panel of each step of the semimorphometric lipid droplets evaluation. Representative expanded blastocyst stained with lipophilic Sudan Black B staining (A). Each expanded blastocyst image was converted to gray scale (B) followed by threshold adjustment (C). Representative stratification of small (< 2 μm; D), large (2 to 6 μm; E) and giant (> 6 μm; F) lipid droplets images. Scale bars = 20 μm.

Semimorphometric evaluation was conducted in order to investigate the total number, total area, and the area per drop of small, large and giant lipid droplets (< 2, 2–6 μm, and >6 μm; respectively). Each image of embryos was converted to gray scale, following threshold adjustment, and using the particle analyzer tool of the Image J 1.4 software, the aforementioned variables were calculated based on the area of each drop size (0 to 3.14, 3.14 to 28.27, and > 28.27 μm^2^; respectively for small, large, and giant lipid drops). The following formula of area (Area = π. r^2^) was used to calculate the area of each drop size based on the ray of each drop (ray = diameter/2). Slighted corrections were conducted when the software merged different drops. A representative panel of each step of the semimorphometric evaluation methodology is presented in the Fig 1.

### Lipid profiles of matrix assisted laser desorption/ionization mass spectrometry (MALDI-MS)

Expanded blastocysts (N = 15 per group) were randomly collected for establishment of embryonic lipid profiles after each treatment. The MALDI-MS technique with 2,5-dihydroxybenzoic acid (DHB) matrix was used. The calibration was performed using eight peptides with known masses as standards. The embryos were washed by pipetting into methanol : H2O (1 : 1) droplets under the stereomicroscope, spotting them on the MALDI plate and allowing them to dry at room temperature. Mass spectra collection was performed in the positive ion mode using an Autoflex III MALDI time-of-flight mass spectrometer equipped with Smartbeam laser technology (Bruker Daltonics). The MS data were acquired in the range from m/z 700 to 1000 by averaging 1200 consecutive laser shots with a frequency of 200 Hz. Embryo spectra were processed (FlexAnalysis 3.3, Bruker Daltonics) for lipid characterization and chemometric analysis using MetaboAnalyt (www.metaboanalyst.ca), as described by Sudano, Santos [20].

### RNA extraction, reverse transcription, and transcript level determination by real-time PCR

Expanded blastocysts (n = 20 per group) were individually collected and stored in small volume of PBS-PVP at -80°C until RNA extraction. The embryos were combined to form pools of five blastocysts, defined as biological replicates and submitted to RNA extraction (n = 4 per group). Total RNA from each blastocyst group was extracted with the PicoPure RNA isolation kit (Life Technologies, Foster City, CA, USA), following the manufacturer's instructions. DNAse treatment was performed on all samples during RNA isolation according to the manufacturer's instructions. The extracted RNA was stored at -80°C. RNA concentration was checked using a Nanodrop instrument (ThermoFischer Scientific, MA, USA), and RNA integrity was evaluated using a Bioanalyzer 2100 (Agilent Technologies, CA, USA) with the use of RICH Pico Chips (Agilent Technologies). All samples analyzed had an RNA integrity number (RIN) ≥ 7. The samples were reverse transcribed for preparation of the cDNA using the High Capacity cDNA Reverse transcription Kit (Applied Biosystems, Foster City, CA, USA), following the manufacturer's instructions. Gene expression analysis was performed using TaqMan Applied Biosystems assays specific for *Bos taurus taurus*. We analyzed the abundance of transcripts for target genes associated with biological processes related to lipid metabolism and embryo quality (for a complete list of gene abbreviations, nomenclature and more information, see S1 Table). Prior to qPCR assays, each sample was subjected to a sequence-specific pre-amplification procedure as follows: 1.25 μL assay mixture (the TaqMan Assay was combined to a final concentration of 0.2 × for each of 96 assays), 2.5 μL TaqMan PreAmp Master Mix (Applied Biosystems, # 4391128) and 1.25 μL cDNA (5 ng/μL). Reactions underwent activation at 95°C for 10 minutes, denaturation at 95°C for 15 seconds, and annealing and amplification at 60°C for 4 minutes for 12 cycles. These pre-amplified products were diluted 5-fold prior to qPCR analysis. The assays and preamplified samples were transferred to an integrated fluidic circuit (IFC) plate. For analysis of gene expression, the prepared sample solution consisted of 2.25 μL of cDNA (pre-amplified products), 2.5 μL of TaqMan Universal PCR Master Mix (2 ×, Applied Biosystems) and 0.25 μL of 20 × GE Sample Loading Reagent (Fluidigm); and assay solution: 2.5 μl of 20 × TaqMan Gene Expression Assay (Applied Biosystems) and 2.5 μl of 2 × Assay Loading Reagent (Fluidigm). The 96.96 Dynamic Array Integrated Fluid Circuit (Fluidigm) chip was used for data collection. After initiation, the chip was loaded with 5 μL of each assay solution and 5 μL of each sample solution and loaded into an automated controller that prepares reactions at nanoliter scales. The thermal cycling qPCR was performed on a Biomark HD System (Fluidigm, South San Francisco, CA, USA) using the TaqMan GE 96 × 96 standard protocol, which consisted of a thermal mixing stage (50°C for 2 minutes, 70°C for 20 minutes and 25°C during 10 minutes), followed by one activation stage (50°C for 2 minutes and 95°C for 10 minutes), followed by 40 cycles of denaturation (95°C for 15 seconds), annealing and primer extension (60°C for 60 seconds). The data were analyzed using the 2^ΔCq^ method and using the median sample from the control group as a calibrator [21]. The geometric mean of the Cq values obtained for the *ACTB*, *GAPDH*, and *PPIA* genes was used as a reference.

### TUNEL (Terminal deoxynucleotyl transferase dUTP nick end-labeling) analysis

Expanded blastocysts (N = 15–18 per group) were fixed in 4% paraformaldehyde for 1 hour at room temperature. After this, they were permeabilized in 0.5% Triton X-100 solution, with 0.1% sodium citrate in PBS for 1 hour. Groups of fixed and permeabilized embryos were subdivided into: positive, negative controls and experimental samples. The positive control was treated with 3U/mL DNase (FPLCpure, Amersham Biosciences) in solution with 400 mM Tris-HCL, 50 mM MgCl_2_ and ultrapure water for 1 hour at 37°C. After washing, the positive control and the samples were incubated in microdroplets of the TUNEL reagent mixture—Kit in Situ Cell Death Detection Kit Fluorescein (Roche, Germany), containing 10% enzyme solution (terminal deoxynucleotide transferase enzyme) with 90% marker solution (dUTP fluorescent conjugate) for 1 hour at 37°C in the dark in a humid camera. The negative control was incubated in microdroplets with marker solution only, in the absence of the enzyme solution. After washing in PBS/PVP solution, controls and samples were mounted on slides containing Hoechst 33342 dye (10 μg/μL) diluted in glycerol for DNA visualization, and for counting the total number of blastomeres for each embryo. This analysis was performed under fluorescence microscopy. Cells that presented FITC-labeled nuclei (green) were considered TUNEL positive cells, that is, they exhibited fragmented DNA. Apoptotic percentage was calculated dividing the number of apoptotic cells by the total number of cells.

### Statistical analysis

Embryo production, cryosurvival, cytoplasmic lipid droplet content, lipid droplets morphometric evaluation, transcripts abundance evaluation, and cell viability data were analyzed by ANOVA using the generalized linear mixed model (GLIMMIX) procedure with the SAS statistical software package (SAS Inst. Inc., Cary, NC, USA), after confirming that data were distributed normally and variances were homogeneous. Treatment, embryo quality (for cryosurvival) and first order interactions were considered fixed effects, whereas replicate was considered to be a random effect. If the results of ANOVA were significant, means were analyzed using the probability of individual differences (PDIFF) test. Logarithmic transformation was applied to qPCR data to improve normality. The data are reported as untransformed least-squares means ± SEMs. Principal component analysis was also used for the qPCR data, and hierarchical clustering of transcript levels was performed using Euclidean distances and Ward linkage to evaluate relationships between samples and features.

For mass spectrometric lipid profile analysis, multivariate and univariate statistical models were used. Ion peak intensities were normalized using the total ion current (TIC) for the spectrum. Missing values were replaced by half of the minimum positive value in the data obtained from the preprocessing procedure. The intensity values of each ion peak across multiple spectra were auto-scaled (mean-centered and divided by the standard deviation of each variable). Principal component analysis (PCA) was performed using MetaboAnalyst 2.0 [22] to identify relationships between variance in the data and differences among different treatment samples. For analyses, a significance level of 5% (P < 0.05) was used.

## Results

### Production of embryos

In our experiments, we tested the effects of negative and positive modulators of ACSL on the development and quality of bovine IVP embryos. 3,393 oocytes were used for the production of 1464 blastocysts. There were no differences (P > 0.05) in the rates of cleavage, embryonic production, and morphological evaluation between the different groups, because of this, were presented just viable blastocysts (grade I and II), as shown in Table 1.

**Table 1:** Effect of long chain Acyl-CoA synthetase modulators during the development of *in vitro* produced bovine embryos: positive (ACS), negative (ACS-), both (ACS±) or none (control).

Group	Oocyte	Cleaved	Cleavage (%)	Blastocysts	Blastocysts/ Oocytes (%)	Blastocysts/ Cleaved (%)
ACS+	829	579	69.8	199	24.0	34.4
ACS-	831	594	71.5	198	23.8	33.3
ACS±	815	581	71.3	190	23.3	32.7
Control	826	600	72.6	221	26.8	36.8

P>0.05

### Vitrification of embryos

The vitrified and subsequently warmed embryos were evaluated for blastocoel re-expansion capacity after 12 hours of further culture. There were no differences (P > 0.05) among treatments or among quality grade in re-expansion rates (Table 2). Embryo hatching rates were evaluated 24 hours and 48 hours after warming. The main effect of treatment and the main effect of embryo quality were observed (P < 0.05) at 24 hours, whereas only the main effect of embryo quality was identified (P < 0.05) at 48 hours after warming. There was no interaction (P > 0.05) effect of treatment and embryo quality in both endpoints (24 and 48 hours), however because of the relevance of the biological results and in order to avoid losing information, the data was not presented and discussed as main effects.

**Table 2:** Cryosurvival of *in vitro* produced bovine embryos treated with long chain Acyl-CoA synthetase modulators: Positive (ACS+), negative (ACS-), both (ACS±) or none (control). Re-expansion rates were recorded with 12 hours after warming while the cumulative hatching rates were recorded with 24 and 48 hours after warming.

Group	Re-expansion 12h (%)			Hatched 24h (%)			Hatched 48h (%)*		
	Grade I	Grade II	Grade III	Grade I	Grade II	Grade III	Grade I	Grade II	Grade III
ACS+	100 (15/15)	95.6 (22/23)	91.3 (21/23)	73.3 (11/15)^A^^a^	65.2 (15/23)^A^^a^	21.7 (5/23)^B^^a^	93.3 (14/15)^A^^a^	91.3 (21/23)^A^^a^	78.2 (18/23)^A^^a^
ACS-	90.3 (28/31)	100 (13/13)	92.8 (26/28)	51.6 (16/31)^A^^b^	38.4 (5/13)^A^^a^^b^	28. 5 (8/28)^B^^a^	83.8 (26/31)^A^^a^	92.3 (12/13)^A^^a^	53.5 (15/28)^B^^b^
ACS±	95.6 (22/23)	91.3 (21/23)	96.5 (28/29)	47.8 (11/23)^A^^b^	34.7 (8/23)^A^^b^	34.4 (10/29)^A^^a^	86.9 (20/23)^A^^a^	65.2 (15/23)^A^^B^^b^	58.6 (17/29)^B^^b^
Control	96.7 (30/31)	96.0 (24/25)	86.2 (25/29)	77.4 (24/31)^A^^a^	60 (15/25)^A^^a^^b^	20.6 (6/29)^B^^a^	96.7 (30/31)^A^^a^	84.0 (21/25)^A^^a^^b^	51.7 (15/29)^B^^b^

^abc^ Within the same column, uncommon lowercase letters differ (P<0.05)

^ABC^ Within the same row for each endpoint (re-expansion 12 hours, hatched 24 hours, and hatched 48 hours), uncommon uppercase letters differ (P<0.05)

* Hatching rates at 48 hours are cumulative

When we analyzed the rating rates of grade I blastocysts at 24 hours, the best results were found in the control and ACS+ groups, which were higher (P < 0.05) than in the ACS- and ACS± groups. In addition, the greatest (P < 0.05) hatching rate at 24 hours of grade II blastocysts was observed on the ACS+ treatment. At 24 hours, embryos of grades I, II and III of the ACS± group presented similar (P > 0.05) hatching rates. However, grade III embryos of the other groups presented lower (P < 0.05) hatching rates compared to those grades I or II.

After 48 hours of incubation, grade III blastocysts treated with ACS-, ACS±, and controls had lower (P < 0.05) hatching rates compared to grade I embryos of their respective groups. Unlike the other treatments, grade III embryos of the ACS+ group presented similar (P > 0.05) hatching rate to embryos of grades I and II of their group and higher (P < 0.05) compared to the grade III embryos of other treatments. Additionally, ACS+ treatment increased the hatching rates of grade II and grade III blastocysts compared to control following 48 hours after warming.

### Lipid droplets content and phospholipid membrane profiles

**Fig 2:**
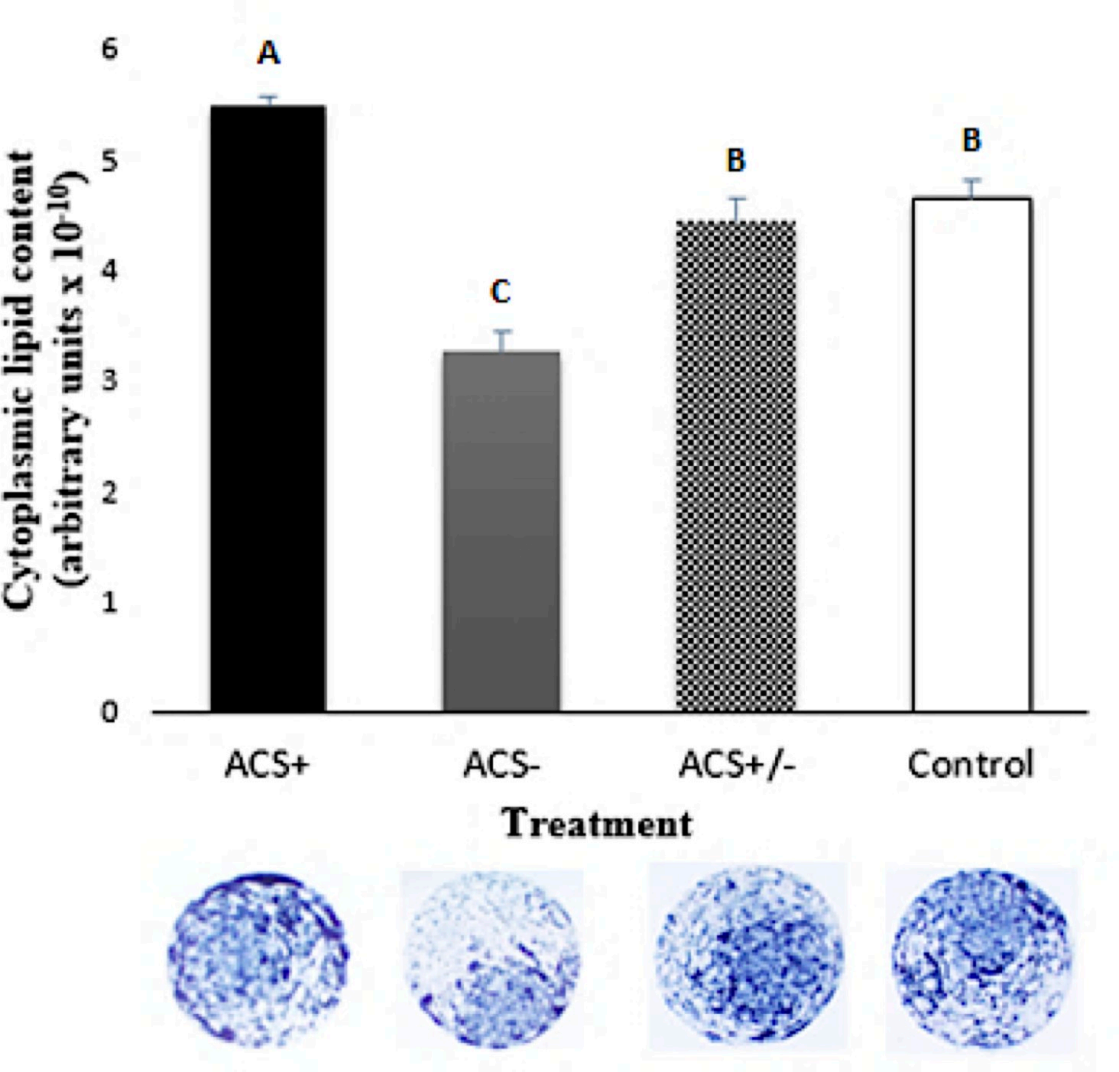
Semiquantitative cytoplasmic lipid content of *in vitro* produced bovine embryos with long chain Acyl-CoA synthetase modulators: positive (ACS+), negative (ACS-), both (ACS±) or none (control). Columns with different letters indicate difference (P <0.05). Sudan Black B staining. N = 18 per group.

Analysis with the lipophilic dye Sudan Black B showed that, when compared to the control group, embryos treated with ACS+ showed higher (P < 0.05) cytoplasmic lipid content, and the embryos treated with ACS- had a reduction (P < 0.05) in lipid content (Fig 2). Additionally, we examined if the positive modulation of Acyl-CoA synthetase through direct regulation of *ACSL3*, mediated by GW3965, accounted for the increase of the lipid content by incubating the embryos also in the presence of Triacsin C. Triacsin C inhibits the acyl-CoA synthetase activity of the ACSL1, 3, and 4 proteins family member [11, 23]. As the *ACSL3* is the only Acyl-CoA synthetase family member that is differentially expressed in both groups and induced by the positive modulation of GW3965, the co-incubation with this inhibitor (Triacsin C) allowed us to separate the direct regulation of *ACSL3* in increasing cytoplasmic lipid content from other Acyl-CoA synthetase family member (ACSL1, 4, 5 and 6). The embryos of the group that received both modulators (ACS±) did not differ (P > 0.05) from control group (Fig 2). The same results were observed in the semimorphometric evaluation of lipid droplets based in the total number of small, large and giant drops (Table 3 and Fig 1). Total area of the drop and the area per drop of small and giant lipid droplets were similar (P > 0.05) among groups. ACS+ blastocysts presented an increased (P < 0.05) total area and area per drop in the large droplets compared to control (Table 3).

**Table 3:** Semimorphometric cytoplasmic lipid droplets evaluation of *in vitro* produced bovine embryos treated with long Acyl-CoA synthetase modulators: Positive (ACS+), negative (ACS-), both (ACS±) or none (control).

Drop Size	Treatment	Drops (N)	Total area (μm^2^)	Area per drop (μm^2^)
**Small (< 2 μm)**	ACS+	154.6±10.2^a^	122.1±11.9	0.86±0.06
	ACS-	98.2±9.3^b^	105.4±11.9	0.76±0.06
	ACS±	123.4±7.2^c^	104.6±11.9	0.83±0.06
	Control	126.6±7.6^c^	102.7±11.9	0.81±0.06
**Large (2–6 μm)**	ACS+	49.8±3.5^a^	467.5±36.2^a^	9.4±0.3^a^
	ACS-	27.0±4.4^b^	244.1±45.3^b^	8.8±0.3^a^^b^
	ACS±	37.4±3.5^c^	325.2±34.2 ^c^	8.7±0.3^b^
	Control	39.8±3.2^c^	347.0±33.3^c^	8.6±0.3^b^
**Giant (> 6μm)**	ACS+	16.0±1.2^a^	9257.8±742.4	581.5±94.9
	ACS-	9.2±1.1^b^	9098.5±664.1	822.8±94.9
	ACS±	13.4±1.5^c^	9659.2±664.1	728.8±84.9
	Control	12.6±1.4^c^	9858.7±664.1	813.1±84.9

^abc^ Within the same column for each drop size, uncommon lowercase letters differ (P<0.05). N = 18 per group.

In the lipid profile analysis, there was no remarkable change in the profiles of membrane phospholipids caused by the different treatments. In the three-dimensional plot of the PCA analysis, sample overlapping was observed with no group individualization among different treatments when the samples were grouped according the identified lipid species abundances (S1 Fig).

### Transcriptional profiles

**Fig 3:**
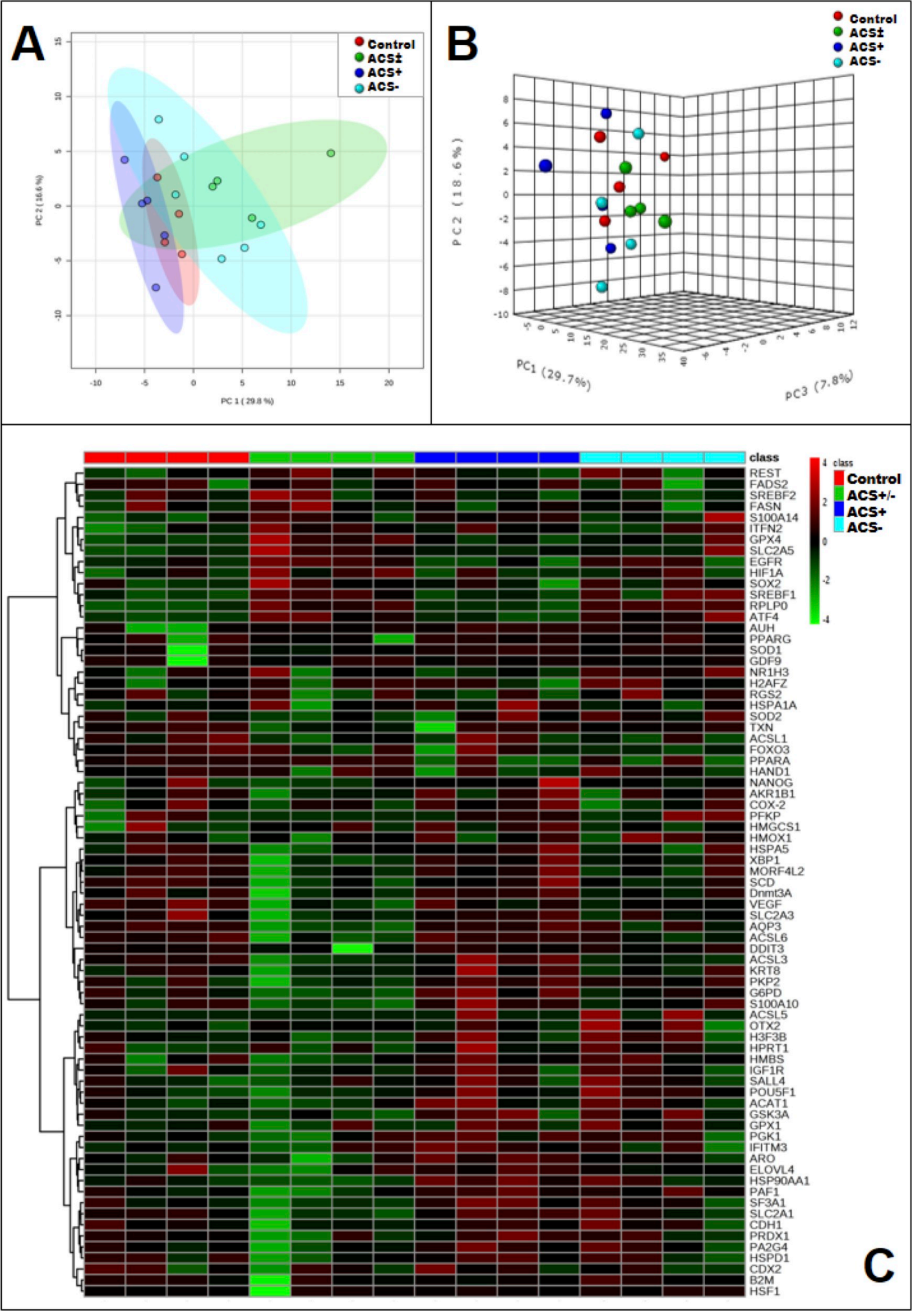
**Two-dimensional (A) and three-dimensional (B) principal component plots and hierarchical clustering of each data set (C) showing transcriptional profiles abundance of *in vitro*–produced bovine embryos with positive (ACS+), negative (ACS-), both (ACS±) or none (control) modulators of long-chain Acyl-CoA synthetases.** N = 4 per group.

No marked individualization of the transcriptional profiles of the gene panel evaluated in the different treatments was observed in the two- and three-dimensional PCA plots (Fig 3A and 3B). Among the 96 genes investigated, 36 genes presented different transcriptional levels (P < 0.05) compared to the control group in at least one of the treatments, of which 18 genes were differentially expressed (P < 0.05) only in the ACS± group (Fig 3C).

**Fig 4:**
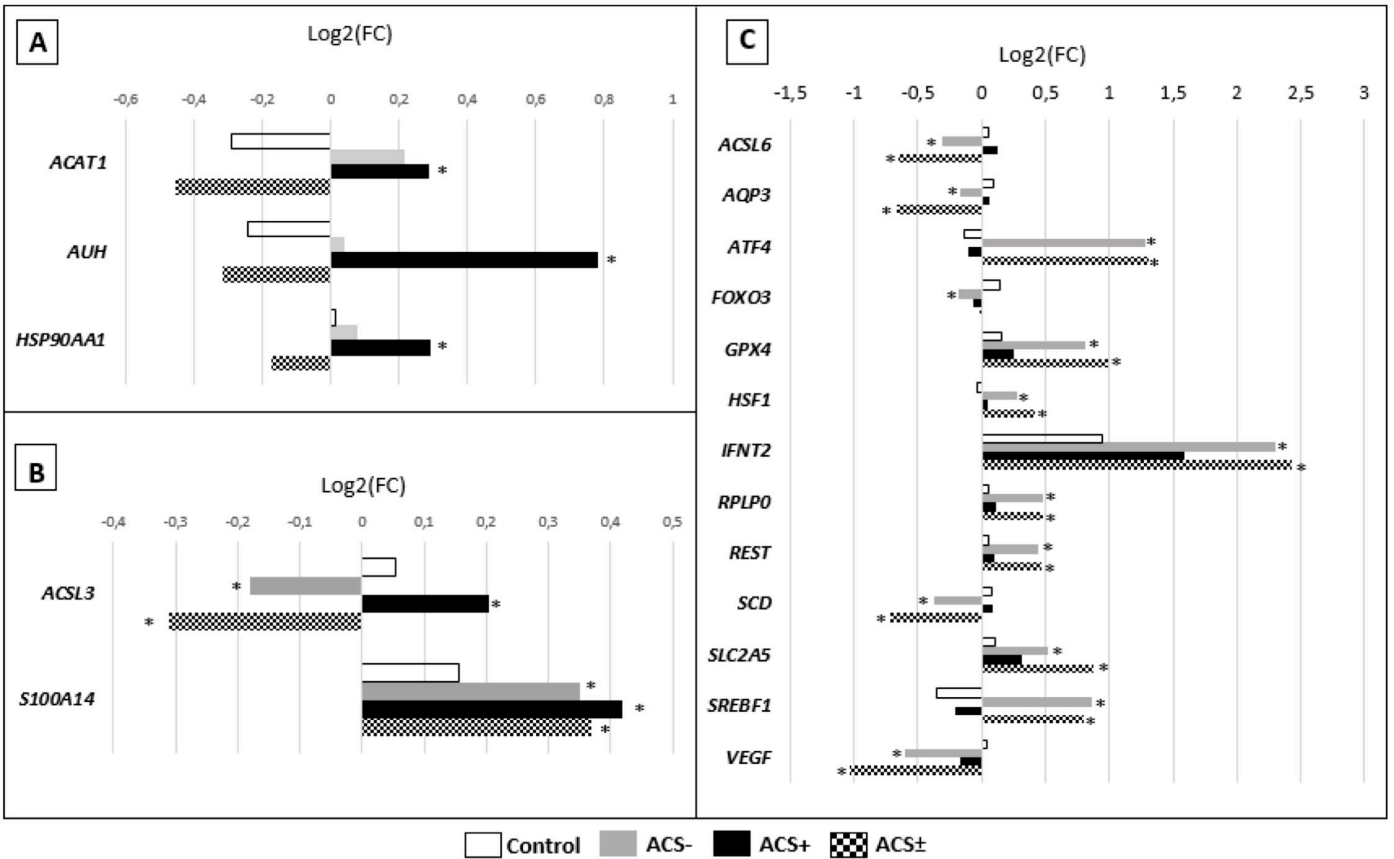
Differentially expressed genes (DEG), compared to control, of *in vitro* produced bovine embryos treated with positive (ACS+), negative (ACS-) both (ACS±) modulators of long chain Acyl-CoA synthetase. (A) ACS+ DEG. (B) ACS+, ACS-, and ACS± DEG. (C) ACS- and ACS± DEG. * represent difference (p <0.05) in relation to the control group. N = 4 per group.

Compared to the control group, genes associated with lipid metabolism were overexpressed (P < 0.05) in the ACS+ (*ACSL3*, *ACAT1*, and *AUH;* Fig 4A and 4B) and in the ACS- (*SREBF1*) group (Fig 4C), or underexpressed (P<0.05) in the ACS- (*ACSL3*, *ACSL6*, *AUH*, and *SCD*), whereas there were no differences (P > 0.05) in the expression of *ACSL1* and *ELOVL* (*1–5*). The expression profiles of genes associated with heatshock were increased (P < 0.05) in both ACS+ (*HSP90AA1*) and ACS- (*HSF1)* compared to the control group. In addition, in the ACS- group, the abundance of gene transcripts associated with oxidative stress (*GPX4*), pregnancy signaling (*IFNT2*), angiogenesis (*VEGFA*), energy metabolism (*SLC2A5*) and transcriptional regulation (*REST* and *RPLPO*) were increased (P < 0.05) compared to the control group, whereas levels of the regulatory genes *AQP3* and *FOX03* were decreased (P < 0.05).

### Embryo cell viability

**Fig 5:**
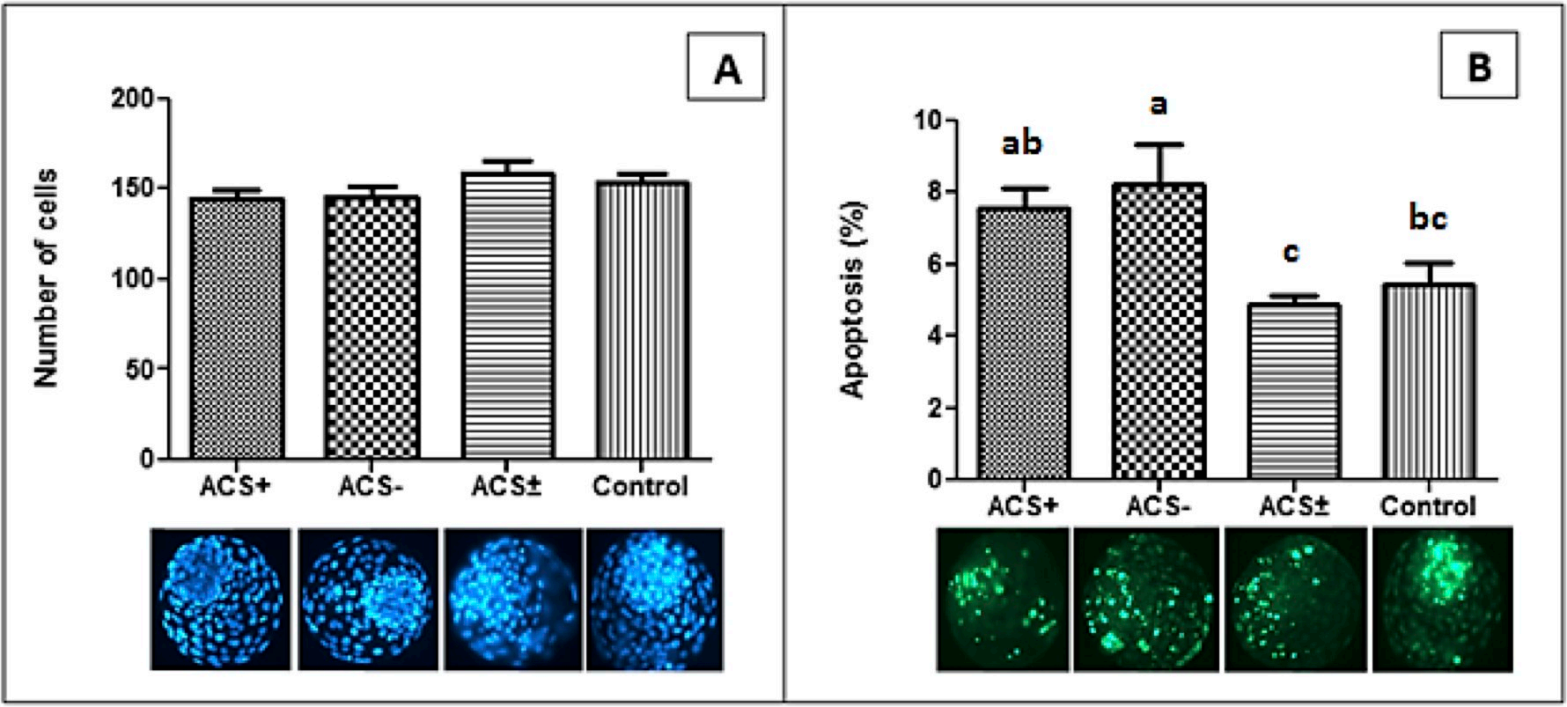
Cell viability analysis of *in vitro* produced bovine embryos with positive (ACS+), negative (ACS-), both (ACS±) or none (control) modulators of long chain Acyl-CoA synthetase. (A) Total number of blastomeres stained with Hoechst 33342 dye. (B) Apoptosis percentage. Columns with different letters differ (P <0.05). N = 15–18 per group.

The total number of blastomeres counted after nuclear staining with Hoechst 33342 did not differ (P > 0.05) among treatments (Fig 5A). In comparison to the control group, the percentage of apoptotic cells with DNA fragmentation was higher (P < 0.05) in the ACS- group (Fig 5B). The other two groups, ACS+ and ACS±, did not differ (P > 0.05) from the control group.

## Discussion

In the present work, we tested the effects of addition of GW3965 hydrochloride (ACS+) and Triacsin C (ACS-), positive and negative modulators of long chain Acyl-CoA synthetase (ACSL), respectively, during the development of IVP bovine embryos. We identified the effectiveness of the treatments in modulating the level of *ACSL3* transcripts, and also the clear impact of this modulation on cytoplasmic lipid droplets content. However, despite the effectiveness of ACS- in reducing cellular lipid deposit, this treatment was not effective in increasing embryonic cryosurvival. Conversely, the use of ACS+ restored the survival rates after vitrification of low quality embryos, making them equivalent to embryos of excellent quality.

Embryo production rates are commonly used as a control endpoint when modifications are made in the culture medium. In our study, the rates of cleavage and production of blastocysts did not differ between groups, evidencing that there were no detrimental or beneficial effects attributed to the use of the modulators on fresh embryo development. The achieved development rates are similar to those commonly described in the literature [3, 24, 25]. Additionally, after morphological evaluation, our results demonstrated that the treatments were not able to significantly alter the degree of quality of the embryos produced when compared to the control group.

To verify the effect of ACS+ and ACS- on total lipid content of the embryos, the lipophilic staining technique of Sudan Black B was used. The results validate the effect initially proposed for both modulators, i.e., the addition of ACS+ to the embryo culture medium was effective in increasing the accumulation of cytoplasmic lipid droplets, whereas a reduction in lipid content was observed in embryos produced with ACS- treatment. The co-incubation of embryos with GW3965 and Triacsin C revealed the direct association between the regulation of *ACSL3* and the cytoplasmic lipid content, i.e. the positive modulation of *ACSL3* accounted for the observed increased cytoplasmic lipid droplets content, by not other Acyl-CoA synthetase family member. Indeed, ACSL3 protein has already been associated with triacylglycerol accumulation and fatty acid uptake in many other cell types [7].

Phospholipids are the most abundant lipids in the membrane of eukaryotic cells, their composition determines crucial physicochemical properties applied in the cryopreservation field, such as fluidity and permeability [26]. At the present work, the lipid profile of the embryos was not altered by the treatments. In this trial only the fatty acid activation was modulated, through *ACSL3* regulation (ACSL3 has a highest affinity for laurate, myristate, arachidonate and eicosapentaenoic [27]), not involving other fatty acid elongases and desaturases that also have great importance on the determination of fatty acids and phospholipids composition. The distinct intracellular location (tissue and intracellular distribution) of each Acyl-CoA synthetases family member has been hypothesized to channel fatty acids at different metabolic fates by activating fatty acids at different subcellular compartments [28]. There are evidences that increased activity of ACSL3 protein is associated with the fatty acid uptake in the placental trophoblasts [7]. These findings also could explain the reason for phospholipid membrane profile remain unaltered by modulators at the present work, i.e., the fatty acids could be directed for triglyceride synthesis and not for altering phospholipid membrane composition. However, an investigation of the regulation of other ACSL family member with its respectively preferred substrate should be conducted to discard their involvement in the determination of the phospholipid membrane composition.

Twelve hours after warming, no differences were found in the rates of re-expansion of the blastocoel in the vitrified embryos, indicating that use of the modulators did not alter either the survival capacity, or the resumption of embryonic metabolic activity, in the initial period after cryopreservation. After 24 hours, however, we surprisingly observed that, grade I embryos, considered as those of high quality that favored pregnancy success after transfer to recipients, presented the best hatching rates in the control and ACS+ groups. Perhaps the resumption of development after cryopreservation in a timely manner could be used as indicative of a greater embryo quality, as frequently used during early embryo (fresh) development [29]. After 48 hours of re-culture, ACS+ was able to restore the hatching rate of grade III blastocysts as like as the results of grade I embryos, resulting in higher hatching rates than the control group. But these findings should be validated with pregnancy rate results, which is considerate the gold standard phenotype, and also on a slow freezing cryopreservation technique in order to verify if the results remain the same.

Although the cytoplasmic accumulation of lipids is commonly associated with a reduction of embryo cryosurvival [1, 30], a cellular and molecular mechanism describing a direct correlation between them is still lacking. In a previous study of our group [20], we have already indicated that just not only the amount lipids affect embryo cryosurvival. When we analyze only ACS+ and ACS- groups, the negative modulation of ACSL3 reduced lipid deposit and do not improve cryosurvival, whereas the positive modulation of ACSL3 resulted in embryos with higher lipid deposit and greater cryosurvival, especially in lower quality embryos. A plausible reason for this contradict result can be explained by the fact that the treatments did not altered total area and the area per drop of giant lipid droplets, especially when larger lipid droplets are considered to be more detrimental for embryo post-cryopreservation survival [1, 31].

Additionally, it is fair to speculate that ACS+ treatment provided beneficial effects for the lower quality embryos by pathways other than lipid metabolism such as energetic metabolism (improving glucose tolerance and insulin resistance by regulating genes involved in glucose metabolism [32]) and inflammatory pathway (inhibiting inflammatory mediators [33]), unfortunately, we did not identify any mRNA transcripts level for genes associated with these pathways in our microfluidic gene panel (the panel was not setup for this pathways).

GW3965 hydrochloride is an agonist specific for LXRα and LXRβ receptors through which it is capable of raising the level of ACSL3 expression. According to Patel, Oza [34] activation of LXR ligands inhibits the induction of NF-κB, an inducible transcription factor. Upon its activation, NF-κB can induce the transcription of multiple genes involved in the cascade pathway of inflammation. The regulation of NF-κB acts not only on the increase of the production of inflammatory cytokines, chemokines and adhesion molecules, but also cell proliferation, apoptosis, morphogenesis and differentiation [35]. It is possible that the embryos of the ACS+ group suffered an inactivation of this pathway, especially grade III embryos, which resulted in a higher rate of cell survival and, consequently, greater survival capacity after cryopreservation. This results also shed light to pathways other than lipid accumulation interfering with embryo survival capacity after cryopreservation.

The various methods of production and culture systems can affect blastocysts cryotolerance and the transcripts expression, which can serve as a sensitive indicator of embryonic quality, and as a marker of developmental competence [36]. In an enzymatic assay conducted by Van Horn, Caviglia [11] in the presence of Triacsin C, there was an inhibition of ACSL1, 3, and 4 isoform activity, but not ACSL5 and 6. In our trials, embryos produced in the ACS- group showed a reduction in the relative abundance of *ACSL3* transcripts and, unlike previous work, we did not find alterations in *ACSL1* levels, whereas there was a reduction in *ACSL6* levels, including in the group treated with both modulators (ACS±). In addition to the reduction in the ACS- group, *ACSL3* expression was stimulated in the ACS+ group, in accordance with results obtained in other studies [7, 13]. In the group that received the addition of both modulators (ACS±), we observed a reduction in *ACSL3* expression compared to the control group. In addition, ACS+ also caused increased expression of other genes related to lipid metabolism, such as *ACAT1*, which converts free intracellular cholesterol to a storage form in cholesterol esters [37], *SCD*, involved in the synthesis of unsaturated fatty acids [38] and *AUH*, more precisely associated with the beta oxidation activity of enoyl-CoA hydratase [39]. Moreover, *Bos taurus taurus* embryos that showed higher cryotolerance also had super-expressed levels of *AUH*, a behavior similar to that identified in the ACS+ group [2].

Induction of HSPs involves a family of heat shock transcription factors (HSF) that bind to the heat shock elements of HSP genes and mediate their transcription [40]. The mechanisms by which *HSF1* is triggered by stress are not entirely understood. It is believed that *HSF1* is repressed by its products—the heatshock proteins—through a mechanism of feedback inhibition [41]. In the ACS- and ACS± groups, there was overexpression of *HSF1* transcripts, which could probably be explained by the low levels of *HSP90AA1* in both groups, unlike in ACS+.

The total number of cells, and the incidence of apoptosis, have been suggested as additional criteria for the evaluation of embryos in order to evaluate the quality and effectively predict their viability [42]. From our results, the total number of blastomeres did not differ between groups. However, embryos from the ACS- group showed higher numbers of cells with DNA fragmentation than the control group. Although it did not affect the rate of production, the use of Triacsin C may have caused embryonic toxicity, since the choice of modulator dosage in the pilot experiment was based only on production rates and morphology of the embryos. In another study, Triacsin C has already demonstrated a dose dependent cell growth inhibitory effect in animal cells [43]. In addition, embryos from the ACS- group showed increased *GPX4* expression and decreased *VEGF* transcript abundance, indicating a possible increase in cellular oxidative stress [44] with consequent developmental impairment [45].

Lipids have different turnover rates in the body. They can be broken down through a series of mitochondrial β-oxidation processes involving Acetyl-CoA, which then enters the tricarboxylic acid cycle to assist the generation of ATP, or alternatively, can be incorporated into triacylglycerols, phospholipids or cholesterol esters [46]. Both distinct pathways require a common initial stage known as activation of fatty acids by ACS. In animal cells the reaction of long chain Acyl-CoA synthetase is the main (probably only) route to supply Acyl-CoA [43]. Thus, we can speculate that the negative modulation of ACSL can lead to the blockade of an important embryonic metabolic pathway, triggering adverse effects that may compromise embryo viability and survival still in the initial period of development.

## Conclusion

In conclusion, the use of GW3965 hydrochloride and Triacsin C during embryonic development effectively modulated ACSL3 and regulated cytoplasmic lipid content without any detrimental effect for embryo yield. Positive modulation of ACSL3 increased the cryosurvival of low quality embryos when submitted to vitrification, such finding could be considered as an alternative for cryopreservation of the larger number of IVP embryos, allowing the cryopreservation of grade I, II and III expanded blastocysts.

## Supporting information

S1 Table Microfluidic panel gene list. Complete list of gene abbreviations, nomenclature and function.
(XLSX)

S1 Fig Three-dimensional PCA plot of lipid profiles of *in vitro* produced bovine embryos with long chain Acyl-CoA synthetase modulators: positive (ACS+), negative (ACS-), both (ACS±) or none (control). N = 15 per group.
(TIFF)
