# Supplementary figures and images for: Modulation of long-chain Acyl-CoA synthetase on the development, lipid deposit and cryosurvival of in vitro produced bovine embryos
Source: PLoS One. 2019 Aug 5;14(8):e0220731. doi: 10.1371/journal.pone.0220731 (PMC6681945; doi:10.1371/journal.pone.0220731)

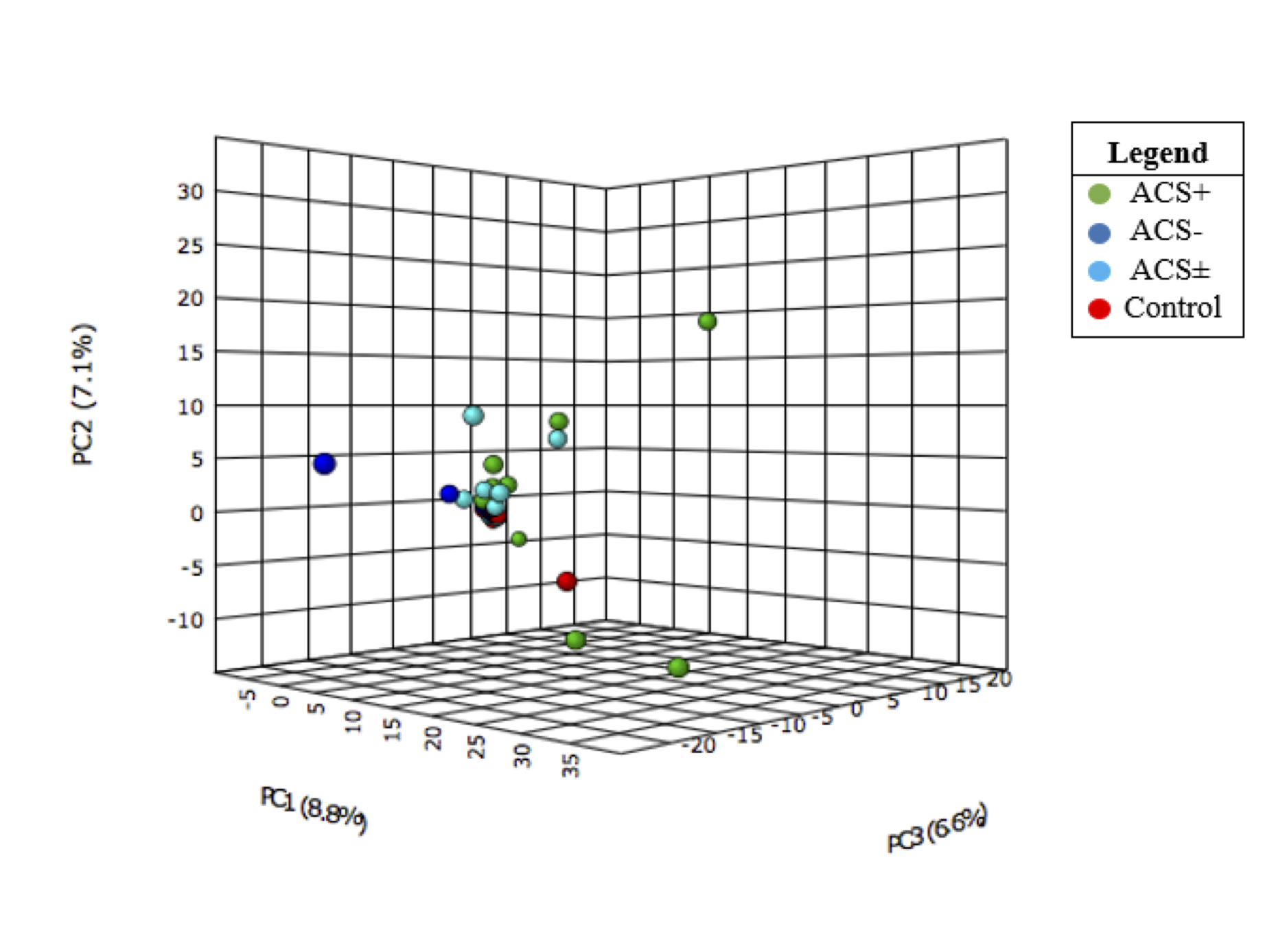

Supplement: S1 Fig — N = 15 per group. (TIFF) [file pone.0220731.s002.tiff]
